# Secreted multifunctional Glyceraldehyde-3-phosphate dehydrogenase sequesters lactoferrin and iron into cells via a non-canonical pathway

**DOI:** 10.1038/srep18465

**Published:** 2015-12-17

**Authors:** Anoop S. Chauhan, Pooja Rawat, Himanshu Malhotra, Navdeep Sheokand, Manoj Kumar, Anil Patidar, Surbhi Chaudhary, Priyanka Jakhar, Chaaya I. Raje, Manoj Raje

**Affiliations:** 1 Institute of Microbial Technology, CSIR, Sector 39A, INDIA-160036 Chandigarh; 2 National Institute of Pharmaceutical Education & Research, Phase X, Sector 67, SAS Nagar, INDIA-160062 Punjab

## Abstract

Lactoferrin is a crucial nutritionally important pleiotropic molecule and iron an essential trace metal for all life. The current paradigm is that living organisms have evolved specific membrane anchored receptors along with iron carrier molecules for regulated absorption, transport, storage and mobilization of these vital nutrients. We present evidence for the existence of non-canonical pathway whereby cells actively forage these vital resources from beyond their physical boundaries, by secreting the multifunctional housekeeping enzyme Glyceraldehyde-3-phosphate dehydrogenase (GAPDH) into the extracellular milieu. This effect’s an autocrine/paracrine acquisition of target ligand into the cell. Internalization by this route is extensively favoured even by cells that express surface receptors for lactoferrin and involves urokinase plasminogen activator receptor (uPAR). We also demonstrate the operation of this phenomenon during inflammation, as an arm of the innate immune response where lactoferrin denies iron to invading microorganisms by chelating it and then itself being sequestered into surrounding host cells by GAPDH.

Lactoferrin, an evolutionarily highly conserved multifunctional glycoprotein present in milk and other body secretions is known for its pivotal role in iron metabolism^1^,^2^,^3^. It has been implicated in multiple physiological functions including; iron homeostasis, cell signalling, immunomodulation, host defence against microbial infections and anti-inflammatory activity^3^,^4^,^5^. Most of these involve, intracellular delivery^4^,^6^, thus making the trafficking of this protein a subject of intense scientific investigation.

Iron sequestration is one of the principal strategies of the innate immune response against microbial infections with lactoferrin as one of the key players^7^,^8^. At the onset of any infection and associated inflammatory reaction, neutrophils recruited to the site of infection secrete iron free lactoferrin (apo-lactoferrin). This Lf efficiently sequesters any iron in the extracellular fluid so as to make it unavailable for utilization by the invading microbes thus hindering their multiplication^9^. This secreted lactoferrin with chelated iron is then cleared by the host system by internalization into the nearby cells, a process so far thought to be limited to occur via interaction with cell surface anchored receptors^10^. On the other hand if this process for internalization of iron loaded lactoferrin was to be additionally facilitated by a soluble and secreted carrier (and not restricted only upon cell surface receptors) it would have the advantage of being able to rapidly internalize larger amounts of iron from far beyond the boundaries of the cells and aiding in the denial of this crucial resource to any invading pathogen.

Glyceraldehyde-3-phosphate dehydrogenase (EC 1.2.1.12), is a ubiquitously present glycolytic enzyme, known to exhibit multiple diverse functions apart from its well characterized role in glycolysis^11^. Earlier we have demonstrated that, GAPDH expressed on the surface of mammalian cells functions as a dual receptor for transferrin as well as lactoferrin^12^,^13^. Role of GAPDH in iron acquisition from host carrier molecules by pathogenic microorganisms has also been reported by several groups^14^,^15^. Apart from its task as a cytosolic enzyme and membrane receptor, GAPDH is also secreted (sGAPDH)^16^ and constitutes a normal component of serum and other body fluids^17^.Recently we also demonstrated that upon iron deprivation cells secrete GAPDH into the extracellular milieu (sGAPDH). This sGAPDH traffics into cells, the iron transport protein transferrin, in an autocrine/paracrine manner utilizing the cell surface molecule uPAR(CD87) and comprises a pool of transferrin receptors distinct from those that are localized on cell surface^18^.

Lactoferrin is structurally and functionally similar to transferrin and our previous studies have demonstrated that lactoferrin and GAPDH interact specifically with high affinity (Kd = 43.8 ± 8.26 nM), in addition, cell surface GAPDH was also demonstrated to bind and traffic lactoferrin to the early endosomal compartment^13^.

Our current study reveals the role of soluble GAPDH in mediating the trafficking of lactoferrin and its associated bound iron into mammalian cells. We also demonstrate that uptake via this pathway is much higher compared to uptake via surface receptors. Finally we also show that, host mammalian cells use this mechanism to sequester iron from invading bacteria as an arm of the innate immune defence mechanism.

## Results

### Soluble GAPDH enhances uptake of lactoferrin into cells

As our previously published work has established that GAPDH on cell surface functions as a dual receptor for both TF and Lf and sGAPDH is a soluble transferrin receptor^19^, we decided to study any possible role for secreted GAPDH in trafficking of Lf into cells. To investigate this, internalization of Lf into a diverse array of primary cells and cell lines was evaluated in presence of extraneously supplemented GAPDH in the incubation medium and compared to the intracellular delivery of Lf through cell surface receptors. This was carried out using quadrant analysis of flow cytometry as described previously^18^ and in methods below. We observed that GAPDH significantly enhanced lactoferrin uptake in most of the cell types studied (1.6 to 6.1 fold more Lf uptake) in comparison to delivery via surface receptors alone (Table 1).

**Figure 1:**
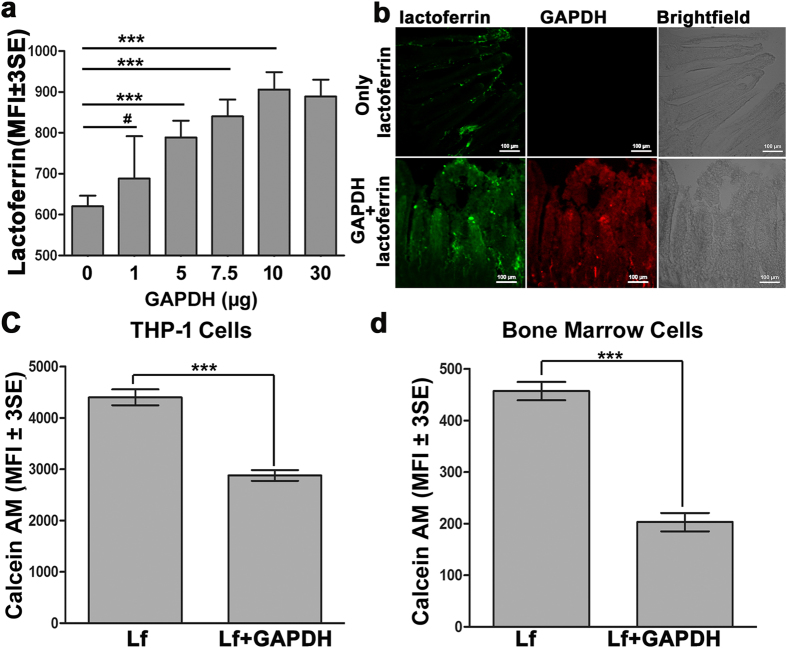
Soluble GAPDH enhances trafficking of lactoferrin and iron into mammalian cells. (**a**) GAPDH enhances Lf uptake by cells in a dose dependent and saturable manner. Aliquots of 5 × 10^5^ J774 cells were allowed to internalize Lf-FITC (5 μg) alone or in presence of increasing concentration of GAPDH in 200 μl FACS buffer at 37 °C for 1 hr. After incubation cells were washed 3X with FACS buffer and treated with pronase (0.1%) to remove residual surface bound ligand. Internalized Lf was quantified using flow cytometry, ^#^p > 0.05, ***p < 0.001, (n = 10^4^ cells). **(b)** GAPDH enhances Lf uptake in isolated mouse duodenum. Ten centimeter duodenal segments of Balb/c mice intestine were washed with PBS and ligated. The lumen was then infused with 5 μg of either Lf-FITC alone (upper panel) or in presence of 10 μg GAPDH-TRITC (lower panel) and incubated for 15 mins at 37 °C. After incubation intestines were extensively flushed with chilled PBS and fixed in buffered formalin over night at 4 °C. Next day small segments of intestine were immersed in 30% buffered sucrose till they sank to bottom of the vial and then tissue was incubated at RT for 30 min before being rapidly frozen in liquid nitrogen. Twenty micron thick cryo sections were cut using a Leica CM 1950 Cryostat, mounted on glass slides and viewed in Nikon A1R microscope using an open pin hole. Experiments were repeated thrice. **(c**,**d**) GAPDH mediated lactoferrin internalization enhances delivery of iron into cells. THP-1 **(c)** and mouse bone marrow cells **(d)** (5 × 10^5^ cells per sample) were incubated with Lf-A647 (10 μg) alone or in presence of GAPDH (20 μg) in 200 μl of PBS for 1 hr at 37 °C. After internalization cells were extensively washed and incubated with 300 nM calcein-AM for 15 min at 37 °C. Labile iron pool of GAPDH incubated samples is significantly higher as compared to control, p < 0.0001, (n = 10^4^ cells).Y-axis in all above experiments represents mean fluorescence intensity (MFI ± 3SE). All experiments were repeated three times, representative graphs are presented.

To check for the dose dependency of this phenomenon, cells were allowed to internalize Lf in presence of increasing concentrations of GAPDH and a dose dependent saturable enhancement in Lf internalization by GAPDH was observed (Fig. 1a). As isolated enterocytes demonstrated an efficient utilization of this pathway in comparison to surface receptors (Table 1) we proceeded to evaluate the physiological significance of this pathway by studying uptake of Lf across mouse intestine duodenum. Fluorescence microscopy of duodenal segments infused with Lf along with GAPDH, demonstrated an enhanced trafficking of lactoferrin into mouse intestinal mucosa in comparison to trafficking of Lf alone (Fig. 1b). A significant amount of Lf was observed to remain bound to the cell surface in absence of GAPDH (probably because of slow internalization via surface receptors), however the uptake was significantly enhanced when incubation was carried out in presence of GAPDH. To confirm our observations at the organism level, mice were orally fed either with Lf-Alexa 633 alone or Lf-Alexa 633 along with GAPDH and imaged using a Perkin Elmer FMT 2500LX *in vivo* imager. Enhanced Lf signal and spatial diffusion, along the alimentary canal was observed in case of mice fed with Lf along with GAPDH as compared to mice fed with Lf alone (Supplementary Fig. S1).

### Soluble GAPDH mediates delivery of lactoferrin bound iron into cells

As Lf is a key protein involved in iron homeostasis, it is imperative to evaluate the effect of enhanced uptake of Lf on iron delivery via this pathway. A significant increase in cellular delivery of iron into THP-1 and bone marrow cells was observed when lactoferrin was internalized along with GAPDH as compared to control cells (Fig. 1c,d).

### sGAPDH and lactoferrin co-traffic into cells

**Figure 2:**
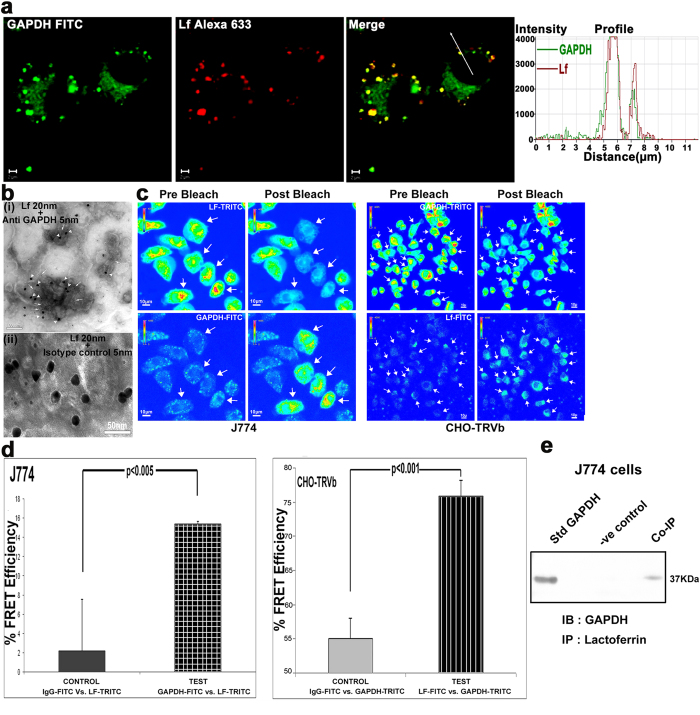
Co-trafficking and interaction between GAPDH and Lf in cells (**a**) Lf and sGAPDH signals are co-localized inside cells. (**b**) (i) Immunogold labeling transmission electron microscopy of purified endosomes from J774 cells demonstrates the co-localization of lactoferrin (20 nm particles indicated with arrowheads) and monoclonal anti GAPDH antibody (5 nm particles marked with arrows). (ii) In place of specific antibody isotype control antibody (5 nm particle) was used. (**c**) Intracellular interaction of Lf and GAPDH was demonstrated by acceptor photobleaching FRET. Bleaching of Lf-TRITC or GAPDH-TRITC (acceptor) signal is accompanied by an increase in the GAPDH-FITC or Lf-FITC (donor) signal in J774 or CHO-TRVb cells respectively (arrows). (**d**) FRET efficiency was calculated from 25 cells in both cases. FRET efficiency was calculated using the following formula, FRET efficienc = [Donor intensity (post-bleach)-Donor intensity (pre-bleach)]/Donor intensity (post-bleach). In control experiments the FRET donor replaced by IgG-FITC. FRET experiments were conducted using a Nikon A1R confocal microscope. (**e**) Interaction between internalized GAPDH and Lf was further confirmed by co-immunoprecipitation from cytosolic fraction of J774 cells. Lf along with biotinylated GAPDH (to distinguish from intracellular GAPDH) was allowed to internalize into J774 cells for 1 hr at 37 °C, cells were then washed and treated with pronase. Lf was immunoprecepitated (IP) using anti-Lf antibody coupled magnabeads. Co-immunoprecipitated biotinylated GAPDH was then detected after immunoblotting (IB) with streptavidin-HRP. Control was run in parallel wherein the cytoplasmic fraction was incubated with isotype IgG coupled magnabeads.

Confocal microscopy demonstrated the co-trafficking of sGAPDH and Lf into cells (Fig. 2a). Immunogold labelling transmission electron microscopy also demonstrated the presence of both molecules in early endosomes purified from cells that had been incubated with sGAPDH and Lf (Fig. 2b). Inter-molecular interaction between the co-internalized Lf and sGAPDH within cells was established using acceptor photobleaching FRET analysis (Fig. 2c,d). Finally intracellular co-trafficking and interaction between the two molecules was confirmed by co-immunoprecipitation (Fig. 2e).

### sGAPDH mediated lactoferrin trafficking into cells involves uPAR and macropinocytosis

**Figure 3:**
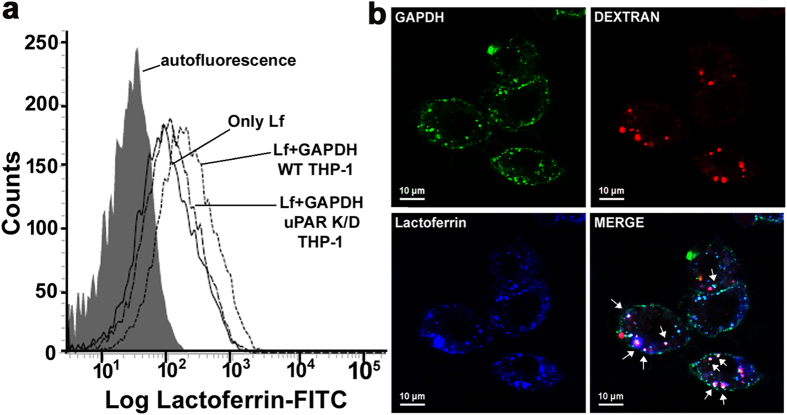
Trafficking of lactoferrin by sGAPDH involves internalization via cell surface uPAR and macropinocytosis (**a**) Lf internalization via GAPDH is decreased in uPAR knock down cells. PMA activated wild type and uPAR knock down THP-1 cells (5 × 10^5^/aliquot) were allowed to internalize Lf-FITC (5 μg) pre-incubated with GAPDH (10 μg) in 200 μl FACS buffer for 1 hr at 37 °C. Cells were washed and treated with pronase (0.1%). Internalized Lf was quantified from 10^4^ cells by flow cytometry and presented as histogram overlay, p ≤ 0.0001. Results were verified by three repetitions of the experiment. (**b**) GAPDH additionally internalizes lactoferrin via the macropinocytosis pathway as demonstrated by co-localization studies in J774 cells. Arrows indicate the strong co-localization of Lf, GAPDH and dextran. Experiment was repeated thrice and representative photograph is presented.

Our earlier published work has demonstrated that sGAPDH is internalized in cells via two different routes involving raft mediated endocytosis as well as macropinocytosis and inhibition of these pathways blocks sGAPDH uptake^18^. We have also established that urokinase type plasminogen activator receptor (uPAR), present on cell surface is a receptor for sGAPDH, facilitating its uptake^18^. To confirm the process utilized by cells for internalization of Lf via sGAPDH, we first evaluated the role of uPAR dependent Lf endocytosis. Utilizing the previously established uPAR knock down THP-1 cell line, we confirmed that depletion of uPAR causes significant decrease in sGAPDH mediated Lf delivery into cells (Fig. 3a). As trafficking of GAPDH into cells has been previously shown to additionally involve macropinocytosis^18^, we checked for that too. Co-localization of Lf and sGAPDH with dextran (a marker of macropinocytosis) confirmed that some trafficking of Lf via sGAPDH also occurs via this pathway (Fig. 3b).

### sGAPDH enhances lactoferrin mediated iron uptake in a murine model of acute peritonitis

**Figure 4:**
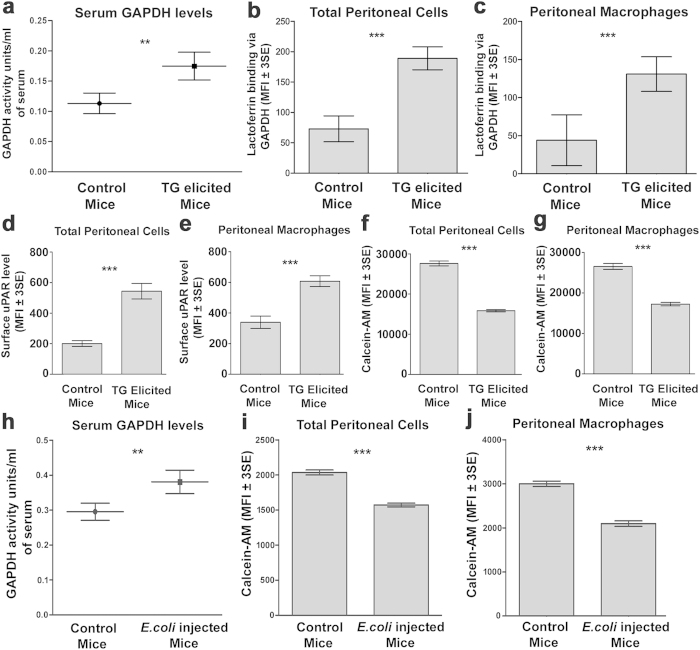
Peritoneal inflammation/infection enhances GAPDH secretion, lactoferrin capture and cellular iron absorption (**a**) TG elicited mice demonstrate enhanced serum GAPDH levels. Female Balb/c mice (8–10 weeks old) were injected i.p with 2.5 ml TG or with PBS (control mice). After 5 days serum was collected and serum GAPDH activity was assayed, p ≤ 0.01, n = 4 each group. (**b,c**) Cells from TG peritonitis induced mice demonstrate enhanced sGAPDH mediated Lf binding as compared to cells from control mice, p ≤ 0.0001, n = 10^4^. (**d,e**) Cells from TG injected mice also demonstrate enhanced surface expression of uPAR, p ≤ 0.0001 n = 10^4^. (**f**,**g**) Calcein quenching assay reveals that peritoneal cells from TG injected mice have higher levels of intracellular iron as compared to control cells, p ≤ 0.0001 n = 10^4^. (**h**) *E.coli* infected mice also demonstrate elevated serum GAPDH levels. Eight to ten week old female Balb/c mice were injected i.p with mid log phase *E.coli* MG1655 (1 × 10^4^) in 500 μl PBS or with PBS alone (Control mice). After 2 days serum was collected and serum GAPDH activity measured, p ≤ 0.01, n = 4 for each group. (**I**,**j**) Host cells from *E.coli* injected mice show enhanced intracellular iron as compared to control cells, p ≤ 0.0001 n = 10^4^. In all flow cytometry analysis the macrophage population was identified using anti F4/80 staining. All experiments were repeated three times and representative graphs are presented.

To investigate, the role of secreted GAPDH in cellular lactoferrin delivery during infection, we utilized a murine model for acute peritonitis (an acute inflammatory condition) as our initial *in vivo* studies had demonstrated that GAPDH induced a >6 fold enhancement of Lf delivery into mouse peritoneal cells (Table 1). In mice with thioglycolate induced acute peritonitis we observed enhanced serum GAPDH (Fig. 4a), suggesting that this may contribute to enhance the Lf sequestering capabilities of host cells. Peritoneal cells and macrophages from TG elicited mice demonstrated increased binding of lactoferrin via sGAPDH compared to cells from control mice (Fig. 4b,c). We also found enhanced surface uPAR expression, which helps in sGAPDH mediated lactoferrin uptake in cells of TG elicited mice (Fig. 4d,e). Finally the iron levels in cells of TG elicited mice were also increased as confirmed by decreased calcein fluorescence (Fig. 4f,g, Supplementary Fig. S2a,b). To further confirm our findings in peritonitis, we used an *E.coli* model of microbial infection. As in case of TG induced peritonitis, here too we found enhanced levels of GAPDH in serum (Fig. 4h) and enhanced sequestration of iron in peritoneal cells isolated from *E.coli* infected mice, a strategy used to deny the availability of this vital element to the invading pathogen (Fig. 4I,j, S2c,d). *E. coli* cells were unable to utilize sGAPDH for binding or internalization of lactoferrin (Supplementary Fig. S2e,f) probably due to lack of uPAR receptors. As the microenvironment in inflammatory conditions is known to demonstrate changes in hydrogen ion concentration and ionic strength we evaluated the capture of Lf by GAPDH over a range of pH and NaCl concentration. At lower pH the capture was observed to be more efficient while ionic strength had no significant effect (Supplementary Fig. S2g,h).

## Discussion

Lactoferrin has long been a molecule of active research due to its pleiotropic properties 3. It has been demonstrated to possess, bacteriostatic/bactericidal and immunomodulatory activities. It also functions as a growth factor and as an enhancer of iron absorption^4^,^20^,^21^. Apart from extracellular iron sequestration most of the physiological effects of Lf are manifested only after its binding and internalization into cells, a process that is dependent upon its interaction with specific cell membrane localized receptors^2^. In literature, very few soluble receptors for large proteins have been identified. In the current study we report that extracellular GAPDH also enhances the internalization of Lf into cells. Significantly more Lf was observed to be delivered by the sGAPDH pathway as compared to cellular uptake via surface receptors alone in a wide variety of cells including those which are known to posses surface anchored lactoferrin receptors (Table 1). As GAPDH has nM range affinity for lactoferrin in solution and also specifically binds to cells^13^,^18^, it is a suitable candidate to function as a soluble receptor for Lf. Our current investigations demonstrate that GAPDH enhances the uptake of Lf into cells, it was also observed to increase its trafficking across the intestinal mucosa in isolated duodenal segments. An enhanced Lf absorption was also observed in mice that were orally fed with Lf along with GAPDH in comparison to mice fed only Lf. Uptake of Lf bound iron by human duodenal biopsies^22^ and in rodent models for treatment of hepatic^23^ and ceacal infections^24^ has been demonstrated recently. The survival of lactoferrin in guts of human neonates and infants has also been documented^2^ and numerous clinical trials are in progress to test the efficacy of orally administered Lf 25.

In concordance to our earlier published results regarding sGAPDH mediated transferrin delivery^18^ here too we observed the uPAR dependent nature of lactoferrin delivery by sGAPDH. Expression of this glycosylphosphatidylinositol anchored membrane protein, also known as CD87, is reported on numerous cells. For example, activated THP1 cells (macrophages) express CD87 while un-activated cells (monocytes) do not^18^. As expected, Lf uptake by sGAPDH was enhanced in case of PMA activated cells but not the un-activated cells (Table 1). Along with enhanced Lf delivery, sGAPDH also increased the delivery of the Lf bound iron into cells. While iron binding is central to some of the biological roles of LF, other properties, including specific interactions with mammalian receptors and microbial components, also contribute to the multifunctional nature of this protein^3^. Earlier workers have documented the increase in Lf levels under conditions of stress such as; iron deprivation, inflammatory disorders, neurodegenerative diseases, infection, etc.^7^. In these conditions it is known to modulate both innate and adaptive immune responses including suppression of pro-inflammatory cytokines, modulation of antigen presentation and affecting phagocytic activity of macrophages^26^. The additional apo Lf secreted during infection efficiently sequesters iron in the vicinity thereby denying its availability to invading microorganisms, once delivered to target cells it also performs additional immunomodulatory activities^21^. Clearance of this additional Lf has till now been believed to be limited to surface receptors (mostly present on macrophages and hepatocytes) only^7^. A recent study has shown that mice pre-injected i.p with GAPDH were protected from septic death, and their serum levels of proinflammatory cytokines were significantly suppressed^27^. Considering that extracellular acidification is a common feature associated with inflammation in several physiological and pathological situations^28^, we evaluated the ability of GAPDH to interact with lactoferrin in the pH 5.8 – 8.0 range. Our analysis revealed that a strong interaction was retained over this range. In fact the capture of Lf by GAPDH was more pronounced at lower pH, a phenomena that would be advantageous in the micro environmental acidosis present in inflammatory loci. We also found that the interaction was independent of the ionic strength.

Our current study is the first to demonstrate the existence of a soluble receptor for delivery of lactoferrin and sequestering of iron into mammalian cells utilizing the higher order multifunctional protein GAPDH^19^. Our current investigations have revealed the presence of elevated levels of sGAPDH in serum of mice with inflammation (both thioglycolate induced as well as due to *E.coli* infection). Host cells obtained from the site of inflammation also had: (i) increased surface expression of uPAR, (ii) demonstrated increased capture of Lf via GAPDH and (iii) higher iron levels. It appears that during infection, more GAPDH is secreted into the extracellular milieu to capture Lf (and the Lf bound iron) for rapid sequestration into surrounding host cells. This foraging of ligand from far beyond the boundaries of the cell membrane, utilizing sGAPDH in an autocrine/paracrine manner, increases the probability of Lf uptake whereby cells are able to maximize acquisition of Lf and iron from the extracellular milieu. Interestingly, even cells that express known surface receptors for Lf were observed internalize more Lf by the sGAPDH route. For example, among the reported receptors of Lf; CD14, LRP and GAPDH are all expressed on macrophage cell surface. Hepatocytes express LRP and asialoglycoprotein receptor while enterocytes are endowed with intelectin^13^,^29^,^30^ (Table 1). The discovery of GAPDH as an additional lactoferrin receptor is unusual given that it is a multifunctional glycolytic enzyme^11^ lacking any similarity to the previously identified receptors. To maintain the availability of Lf or any other bioactive entity for that matter, at an optimal level, living organisms need to develop well orchestrated homeostatic mechanisms to regulate the absorption and transport both at the cellular and systemic levels. Although unicellular organisms face similar challenges, vertebrates have evolved distinct, highly specialized mechanisms for this purpose involving specific receptor mediated uptake^29^. Glycolytic enzymes would have evolved during the early development of unicellular organisms. As these are expressed in high copy number and are relatively conserved in sequence & function across species, they are ideal candidates for endowment with multiple functions especially those that may be have to be called upon in times of acute emergencies such as infection/inflammation. Evolution of GAPDH as a receptor for lactoferrin uptake may have occurred before more specific receptor molecules (CD14, LRP, etc.) evolved^31^ for this specific purpose. As primitive unicellular organisms would be present in a fluid medium there would be obvious advantages to evolve a secreted form of this receptor in addition to one localized on the surface, thereby providing an additional dimension for acquisition of target molecules. While iron sequestration into host cells can be an effective defense against extracellular bacterial infections conversely it may favor intracellular pathogens by making more iron available for them^32^. One could speculate that infectious microorganisms that reside inside mammalian cells may have evolved to take advantage of this host iron sequestering mechanism. Interestingly some intracellular pathogens notably *M. tuberculosis* also utilize their surface GAPDH as a receptor for host iron carrier proteins^15^.

The widespread biological functions of Lf have led to investigations for the therapeutic applications of lactoferrin. It is classified by the FDA as a GRAS (generally recognized as safe) product and is widely used as an additive in infant formulas, food supplements, and other health-benefit products. Various clinical trials conducted to evaluate the efficacy of lactoferrin have demonstrated the feasibility for use of this multifunctional glycoprotein as a pharmaceutical agent in diverse medications^2^,^25^,^26^,^33^. Our finding could be relevant in pathological conditions involving highly elevated levels of GAPDH that have been found in clinical samples from patients of iron deficiency^34^, infection and cancer^35^. Attempts are in progress to utilize it for targeted clinical therapy to deliver therapeutic molecules^36^ and in this context our studies on sGAPDH could also play a role.

## Methods and Materials

### Cell Lines, primary cells and materials

J774 (mouse macrophage cell line) was procured from ECACC. CHO-TRVb (which lacks several surface Lf & transferrin receptors) was kindly provided by Prof. T.McGraw, Weil Cornell^13^. All other cell lines were obtained from National Centre for Cell Sciences, Pune, India. THP1 cells were utilized either directly (unactivated) or after activation into macrophages by treatment with 12.5 ng/ml of PMA for 24 hours. All primary cells: spleen & peritoneal macrophages, enterocytes, hepatocytes, bone marrow cells (from Balb/c mice) and human peripheral blood lymphocytes were purified as described previously^18^. Cells were maintained in RPMI-1640 medium supplemented with 10% foetal calf serum. Viability of all cells was regularly monitored before experiments and was typically >99% as described earlier^18^. All animal handling protocols were as approved by the institutional animal ethics committee (see statement of ethical standards below). Blood for isolation of lymphocytes was obtained from normal healthy volunteers as per relevant institutional procedures and approvals. *E.Coli* MG1655 was kindly gifted by Dr. S. R. Chaudhuri^37^. Rabbit muscle GAPDH and human lactoferrin were obtained from Sigma. GAPDH and lactoferrin were conjugated to biotin and fluorochromes as described previously^18^. Rabbit polyclonal antibody against Lf was raised and validated by western blot using standard laboratory protocols^13^.

### Silencing of uPAR

A stable THP1 cell line in which knockdown of total cellular uPAR as well as depletion of surface expression had been established previously^18^ was used to study the role of this molecule in Lf trafficking via sGAPDH.

### Flow Cytometric analysis

All FACS analysis was performed essentially as described earlier^12^,^18^. Briefly, 5 × 10^5^ cells were incubated either with 10 μg of GAPDH-FITC and/or with 5 μg LF-Alexa633 in 200 μl of FACS buffer (20 mM HEPES pH 7.4, 150 mM NaCl, 1 mM CaCl_2_, 1 mM MgCl_2_, 5 mM KCl and 5% fetal calf serum). Cell surface uPAR was detected by staining with 1 μg anti uPAR antibody (R&D Systems) followed by incubation with 1:250 diluted anti rat-FITC (Sigma). Macrophage population from peritoneal lavage was identified with anti F4/80-APC staining. Data was acquired using either a FACS Verse or Accuri C6 flow cytometers (BD) from 10^4^ cells of each sample and presented as mean fluorescence intensity (MFI) ±3SE.

### GAPDH supplementation and sGAPDH enriched culture supernatant enhances lactoferrin trafficking into cells

To study lactoferrin internalization, primary cells or cells from confluent cultures were incubated with 5 μg lactoferrin-FITC along with increasing concentrations of GAPDH in 200 μl of FACS buffer for 1 hr at 37 °C. In order to quantify only the internalized Lf all residual surface bound (non internalized) Lf was digested by treating cells with pronase. For each sample, internalized Lf was quantified in 10^4^ cells using flow cytometry.

### Relative trafficking of lactoferrin by sGAPDH and via surface receptors

Relative trafficking of Lf by sGAPDH as compared to cell surface anchored receptors was performed exactly as described previously^18^. Briefly, cells were incubated at 37 °C for 30 minutes with either; (i.) only lactoferrin-Alexa633 (5 μg), (ii.) only GAPDH-FITC (10 μg) or (iii.) both (i.) & (ii.) in 200 μl of FACS buffer and analyzed by flow cytometry for internalization of lactoferrin and GAPDH. A two-color quadrant plot was established by plotting events for internalized Lf and GAPDH signals along the x and y axes respectively. Autofluroscence in either channel determined the threshold of labeling. The lower right quadrant represents cells that are positive for only lactoferrin (uptake only via surface receptors) while cells in the upper right quadrant represent cells that have co-internalized both signals (Lf internalization via surface receptors + internalization mediated by sGAPDH). A comparison of, signal intensity (MFI) of Lf between the two quadrants provides information on the extent of Lf internalization by the surface receptor or surface receptor + sGAPDH receptor route^18^. For *in vivo* uptake BALB/c mice were injected i.p with fluorescent labeled Lf with and without GAPDH supplement. After 1 h cells from peritoneal lavage were collected and analyzed as above. Peritoneal macrophages were identified by anti F4/80 staining (Data presented in Table 1).

### Lactoferrin uptake across mouse duodenum

Uptake of Lf and GAPDH across isolated mouse duodenal segments was performed by modification of a protocol described previously^38^. Ten centimeter sections of duodenum from Balb/c mice were dissected out and flushed with PBS. Both ends of the intestine were then ligated and 5 μg of either Lf-FITC alone or along with of 10 μg GAPDH-TRITC in 200 μl PBS was injected into the lumen and the whole preparation incubated for 15 minutes at 37 ^o^C in dark. After incubation intestines were extensively flushed with chilled PBS and fixed in buffered formalin (SIGMA) over night at 4 ^o^C. Next day small pieces of intestine were immersed in 30% buffered sucrose till they sank to bottom and then held at RT for 30 min before being rapidly frozen in liquid nitrogen. A Leica CM 1950 Cryostat was used to cut 20 μ cryo-sections which were mounted on glass slides and viewed in a fluorescence microscope.

### Lactoferrin-iron uptake by calcein fluorescence quenching

Lactoferrin mediated iron uptake was assessed by modification of calcein-AM quenching assay^15^. Aliquots of 5 × 10^5^ cells were incubated with Lf-Alexa647 (10 μg) along with GAPDH (10 μg) in 200 μl PBS for 1 hr at 37 °C, in parallel controls GAPDH was omitted. Cells were then washed and loaded with 300 nM calcein-AM (Sigma) for 15 min at 37 °C. The intracellular labile iron pool was evaluated by quantifying the quenching of calcein fluorescence using flow cytometry. Data are plotted as MFI ± 3SE of 10^4^ cells.

### Co-localization of soluble GAPDH and lactoferrin in cells and purified endosomes

J774 cells grown on glass coverslips were incubated with lactoferrin Alexa 633 + GAPDH-FITC at 37 °C for 15 minutes and then imaged using a confocal microscope (LSM 512 META) using 1Airy unit (AU) aperture as described previously^30^. To visualize simultaneous presence of both Lf and GAPDH in endosomes by electron microscopy, J774 cells (5 × 10^7^) were allowed to internalize the two proteins as above and endosomes were prepared as described previously^13^. Purified endosomes were adsorbed onto carbon coated nickel grids and blocked with 2% BSA. GAPDH was detected using rabbit anti-GAPDH along with 5 nm gold conjugated anti-rabbit-IgG (Sigma). Biotinylated Lf was detected with 20 nm Streptavidin gold conjugate (Sigma). Grids were negatively stained with phosphotungstic acid and uranyl acetate before observation in a JEOL JEM 2100 transmission electron microscope. Internalization of lactoferrin-GAPDH via macropinocytosis was confirmed by confocal co-localization microscopy exactly as described previously^18^. Briefly 2 × 10^5^ cells cultured on LabTek® chambered coverslips were blocked with 2% BSA for 30 min at 4 °C and then incubated in 200 μl serum free media containing 5 μg Lf-A647, 10 μg GAPDH-FITC and 2 μg Dextran-TRITC (Molecular Probes), for 30 min at 37 °C. After washing with buffer, cells were fixed in 2% paraformaldehyde and imaged in a confocal microscope (Nikon A1R) using 1 AU aperture.

### Confirmation of sGAPDH-Lf interaction by co-immunoprecipitation and Foster Resonance Energy Transfer (FRET) analysis

Co-immunoprecipitation (Co-IP) of Lf and biotinylated GAPDH (to discriminate from pre existing intracellular GAPDH) from cells was performed as previously described^15^. Briefly, 1 × 10^7^ cells were allowed to co-internalize, 1000 μg biotinylated GAPDH along with 500 μg Lf in 500 μl PBS at 37 ^o^C for 30 minutes before preparation of cytosolic fraction. For this, cells were washed thrice with PBS, pronase treated and then homogenized in 1 ml of homogenization buffer (20 mM Tris-Cl, 1 mM EDTA, 0.25 M sucrose, pH7.4) at 4 °C. The nuclear fraction was removed by centrifugation at 800 g for 10 min. Supernatant (cytosolic fraction) was utilized for Co-IP. Internalized Lf was immunoprecipitated using rabbit anti-Lf coupled magnabeads® (Pierce) and the presence of co-internalized biotinylated GAPDH was detected with streptavidin-HRP on western blots. Control was run in parallel wherein the cytoplasmic fraction was incubated with Isotype control IgG coupled magnabeads. Interaction of Lf and GAPDH inside the cells was also confirmed by acceptor photobleaching FRET analysis as described earlier^30^. Bleaching of Lf-TRITC or GAPDH-TRITC (acceptor) signal is accompanied by an increase in the GAPDH-FITC or Lf-FITC (donor) signal in J774 or CHO-TRVb cells respectively.

### Induction of peritonitis

Murine model of peritonitis was induced as described previously^39^. Briefly 8–10 week old Balb/c mice were injected i.p. with 2.5 ml thioglycolate (TG) (BD biosciences) or with 1 × 10^4^ *E.coli* MG1655 in 500 μl sterile PBS. Control mice were injected with only PBS. After 5 days (2 days in case of *E.coli* infection) serum was collected and peritoneal cells were harvested in 5 ml chilled PBS. Lf binding via GAPDH, surface uPAR expression and intracellular iron levels were assessed as described above. Serum GAPDH activity was assessed as described above.

### GAPDH mediated lactoferrin binding and uptake on *E.coli* cells

Briefly 2 × 10^8^ *E.coli* cells were blocked with 2% BSA in 100 μl PBS for 1 hr on ice and incubated with Lf-A647 (20 μg) alone or along with GAPDH (40 μg) in 100 μl PBS. Cells were then incubated at 4 °C (for assessment of binding) and 37 °C (for evaluation of uptake) for 1 hr. Cells were pronase treated in uptake experiments as described above to remove surface bound lactoferrin. Data were plotted as mean fluorescence intensity ± 3SE of 10^4^ cells.

### Binding assay to evaluate effect of pH and ionic strength on GAPDH-Lf interaction

To evaluate the effects (if any) of pH and ionic strength on the capture of Lf by GAPDH we used a solid phase binding assay essentially as described previously^30^. briefly, polystyrene wells were coated overnight at 4 °C with 1 μg/well of GAPDH in 20 mmol/L Tris-Cl buffer (pH 8.3) and blocked using 2% casein for 2 hr at 4 °C. Coated wells were then incubated for 2 hr with 3 μM biotinylated lactoferrin in phosphate buffer adjusted to the indicated pH. Bound lactoferrin was then detected with streptavidin-HRP and TMB-H_2_O_2_ (SIGMA). Absorbance at 450 nm was recorded using a plate reader. Interaction between the two proteins in varying at ionic strength was also studied similarly, wherein biotinylated lactoferrin was incubated in phosphate buffer (pH 7.4) with increasing concentrations of NaCl (0–500 mM) and bound lactoferrin measured as above.

## Additional Information

**How to cite this article**: Chauhan, A. S. *et al.* Secreted multifunctional Glyceraldehyde-3-phosphate dehydrogenase sequesters lactoferrin and iron into cells via a non-canonical pathway. *Sci. Rep.* **5**, 18465; doi: 10.1038/srep18465 (2015).

## Supplementary Material

Supplementary Information

## Figures and Tables

**Table 1:** Enhancement of Lactoferrin uptake in cells by GAPDH.

Cell type	Fold change in Lactoferrin MFI of cells internalizing both GAPDH and Lf vs. only lactoferrin +ve cells
Mouse Enterocytes	5.52*
Mouse Hepatocytes	4.28*
Mouse Bone Marrow cells	3.7*
Mouse Spleen Macrophages	2.1*
Mouse Peritoneal Macrophages (*Ex vivo*)	1.17*
Mouse peritoneum cells (*In-Vivo)*	6.1*
Human Lymphocytes	1.27*
J774	2.62*
PMA activated THP1	2.07*
Non activated THP1	No significant uptake of Lf
CHO	4.95*
CHO-TRVb	2.83*
K562	1.6*
Neuro-2A (N2A)	4.02*

Relative trafficking of Lf by sGAPDH as compared to surface receptors. 5 × 10^5^ cells were incubated at 37 °C for 30 minutes with either; (i.) only lactoferrin Alexa-633 (5 μg), (ii.) only GAPDH-FITC (10 μg) or (iii.) both (i.) & (ii.). After internalization cells were washed, pronase treated and analyzed by FACS. A two-color quadrant analysis was done to see relative trafficking as described in methods, *p < 0.001 compared to Lf internalized via surface receptors only. n = 10^4^ cells in each case.
