# Supplementary material for: Secreted multifunctional Glyceraldehyde-3-phosphate dehydrogenase sequesters lactoferrin and iron into cells via a non-canonical pathway
Source: Sci Rep. 2015 Dec 17;5:18465. doi: 10.1038/srep18465 (PMC4682080; doi:10.1038/srep18465)
Supplement: Supplementary Information [file srep18465-s1.pdf]

Supplementary Material

Secreted multifunctional Glyceraldehyde-3-phosphate dehydrogenase sequesters lactoferrin and iron into cells via a non-canonical pathway

Anoop S. Chauhan[1, ‡], Pooja Rawat[1, ‡], Himanshu Malhotra[1], Navdeep Sheokand[1], Manoj Kumar[1], Anil Patidar[1], Surbhi Chaudhary[1], Priyanka Jakhar[1], Chaaya I.Raje[2] and Manoj Raje[1]

[1] Institute of Microbial Technology, CSIR, Sector 39A, Chandigarh, INDIA-160036.

[2] National Institute of Pharmaceutical Education & Research, Phase X, Sector 67, SAS Nagar, Punjab, INDIA-160062.

[‡]Both these authors contributed equally to the manuscript.

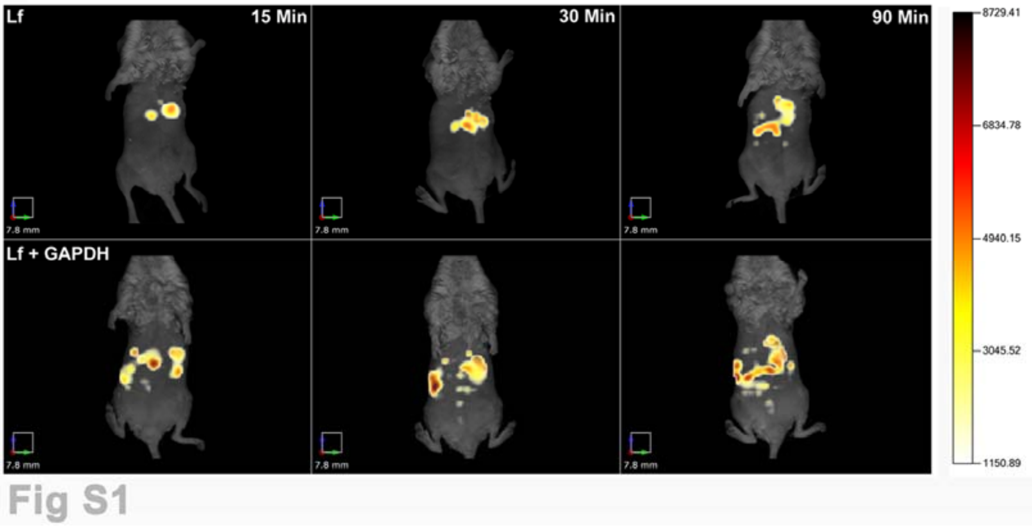

Supplementary Figure S1. Lactoferrin uptake in orally fed mice is enhanced by sGAPDH. GAPDH supplementation enhances intestinal absorption of Lf in mice, Overnight starved, 10-12 week old female Balb/c mice were orally fed with, either 500µg Alexa-647 labeled Lf (upper panel) alone or labeled Lf along with 1mg GAPDH (lower panel). After feeding, mice were imaged at 15, 30 and 90 min intervals in a Perkin Elmer FMT 2500LX *in vivo* imager. Results are representative of 3 independent experiments.

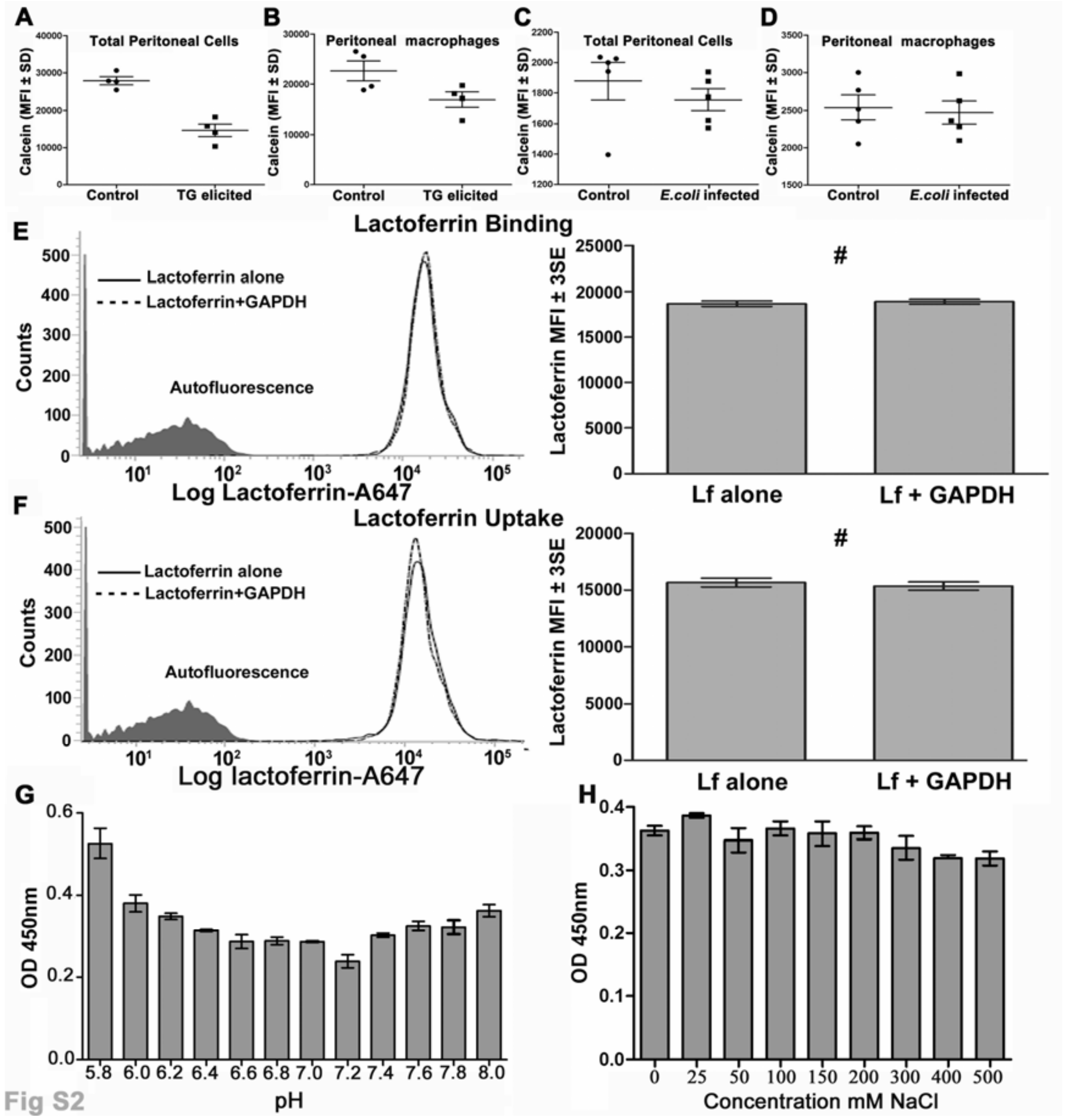

Supplementary Figure S2. Iron levels in cells isolated from peritonitis induced animals are elevated. (A - D) Calcein quenching assay reveals that iron levels in peritoneal cells and macrophages isolated from TG elicited peritonitis **(A & B)** and *E.coli* infected **(C & D)** mice are elevated. Each group consists of 4 mice. *E.coli* cells are unable to utilize the sGAPDH mediated lactoferrin uptake pathway. **(E&F)** Bound or internalized Lf was quantified by flow cytometry from 10^4 cells incubated with Lf-A647alone or along with GAPDH. Data is presented as overlay (left) with bar graph of the same experiment presented on right. $p \geq 0.05$. **(G&H)** Lactoferrin capture by GAPDH immobilized in polystyrene wells at different pH and ionic strength. A strong interaction was observed at lower pH **(G)** while increasing ionic strength did have any significant affect **(H)**. All experiments were repeated three times and representative graphs are presented.
